# Human Meibum Age, Lipid–Lipid Interactions and Lipid Saturation in Meibum from Infants

**DOI:** 10.3390/ijms18091862

**Published:** 2017-08-28

**Authors:** Samiyyah Sledge, Collin Henry, Douglas Borchman, Marta C. Yappert, Rahul Bhola, Aparna Ramasubramanian, Ryan Blackburn, Jonathan Austin, Kayla Massey, Shanzeh Sayied, Aliza Williams, Georgi Georgiev, Kenneth N. Schikler

**Affiliations:** 1 Department of Ophthalmology and Visual Sciences, University of Louisville, Louisville, KY 40202, USA; samiyyah.sledge@louisville.edu (S.S.); collin.henry@louisville.edu (C.H.); rbhola@choc.org (R.B.); a0rama09@exchange.louisville.edu (A.R.); ryan.blackburn@louisville.edu (R.B.); jsaust02@gmail.com (J.A.); kayla.massey@bison.howard.edu (K.M.); shanzehsayied@gmail.com (S.S.); aliza.williams@centre.edu (A.W.); 2 Department of Chemistry, University of Louisville, Louisville, KY 40292, USA; mcyappert@louisville.edu; 3 Division of Ophthalmology, Children’s Hospital of Orange County, Orange, CA 92868, USA; 4 Model Membranes Lab, Department of Biochemistry, Faculty of Biology, St. Kliment Ohridski University of Sofia, Sofia 1164, Bulgaria; g.as.georg@gmail.com; 5 Department of Pediatrics, University of Louisville, Louisville, KY 40202, USA; knschi01@exchange.louisville.edu

**Keywords:** age, dry eye, FTIR, lipids, meibum, NMR, spectroscopy

## Abstract

Tear stability decreases with increasing age and the same signs of instability are exacerbated with dry eye. Meibum lipid compositional changes with age provide insights into the biomolecules responsible for tear film instability. Meibum was collected from 69 normal donors ranging in age from 0.6 to 68 years of age. Infrared spectroscopy was used to measure meibum lipid phase transition parameters. Nuclear magnetic resonance spectroscopy was used to measure lipid saturation. Increasing human meibum lipid hydrocarbon chain unsaturation with age was related to a decrease in hydrocarbon chain order, cooperativity, and in the phase transition temperature. The change in these parameters was most dramatic between 1 and 20 years of age. Meibum was catalytically saturated to determine the effect of saturation on meibum lipid phase transition parameters. Hydrocarbon chain saturation was directly related to lipid order, phase transition temperature, cooperativity, changes in enthalpy and entropy, and could account for the changes in the lipid phase transition parameters observed with age. Unsaturation could contribute to decreased tear film stability with age.

## 1. Introduction

Tear lipids, mostly from the Meibomian gland and a minor amount from sebaceous glands [1], may be important for tear stability [2,3,4]. Changes in tear film lipid composition with age could give us insights into lipid compositional-functional relationships with dry eye. For instance, the signs of dry eye such as decreased breakup time and increased blink rate, are exacerbations of the same signs observed with aging [1,2,3,4,5,6,7,8,9,10,11,12,13,14,15]. The spontaneous blink rate of adults is as much as 20 times per minute, much higher than that of infants which blink less than one time a minute [15]. The spontaneous blink rate is related to the tear break-up time. Tear break-up time is as high as 35 s in infants and decreases to 8–16 s in adults. Tear break-up time is even lower (5 s) in adults with Meibomian gland dysfunction [16,17,18,19,20,21,22].

This project is an extension of previous nuclear magnetic resonance (NMR) [23,24] and Fourier transform infrared (FTIR) [1,24,25,26,27,28] spectral studies relating age with meibum composition, structure, and function. Using deuterated chloroform as a solvent rather than deuterated cyclohexane used previously [23,24], in the current study, the double bond resonance assigned to cholesterol was resolved and quantified from the double bond resonance associated with hydrocarbons using a 700 MHz NMR spectrometer. The 700 MHz NMR spectrometer is more powerful than the 500 MHz NMR spectrometer used previously [23,24]. Furthermore, catalytic hydrogenation was used to examine the relationships between hydrocarbon chain order and the level of saturation. This was an improvement over the previous study where only native meibum was compared with meibum that was 100% saturated [25], a level that is not physiological. This study provides insights into how the increase in meibum lipid saturation can be related to the observed decrease in tear film stability with age.

## 2. Results

### 2.1. Donor Demographics

Meibum was obtained from donors that did not have observable signs of Meibomian gland blockage or any general complaint of dry eye. Meibum for the catalytic saturation study was pooled from six Caucasian females 23, 24, 28, 32, 66, and 68 years old; a 29-year-old Caucasian male; and a 32-year-old African American female. Meibum for the saturation-age NMR study were obtained from the following donors: CM0.6, CM01, CM01, BM04, CM01, BM01, CM01, CF1.4, BM1.6, CM1.8, HF2, CM02a, CF02, CM02, CM02, AM03, CM03, CM03, CM04, CM04, CM04, CF04, CM04, CF05, CM05, CM05, CF05, XM08, XM08, CF8.5, BM10, CF11, CF11, CF13, CM14, CM17, BF19, CF20, CM22, CF23, CF24, CF24, CM25, CF26, CF27, CF28, HF29, CM29, CM31, CM31b, B32F, CF36, HF37, BF38, BM39, CF43, CM55, CM59, CM62, CF66, and CF68. The sample key is: C = Caucasian; H = Hispanic; A = Asian; B = African American; X = unknown race; F = female; M = male; and numbers are age. Meibum was collected only once from each donor.

### 2.2. H-NMR Spectroscopy

**Figure 1:**
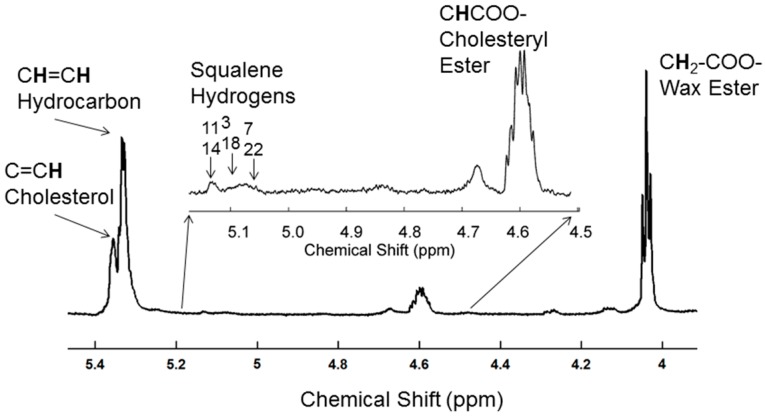
Typical proton nuclear magnetic resonance (^1^H-NMR) spectrum of meibum from a five-year-old Caucasian male.

Average proton NMR (^1^H-NMR) spectra of human meibum were typical of meibum collected from a 700 MHz spectrometer (Figure 1). Band assignments were made based on previous ^1^H and carbon 13 NMR studies [23,29]. The largest resonance in this region was observed at 5.32 ppm with a shoulder at 5.35 ppm assigned to protons of the *cis* =CH moieties from hydrocarbon chains and to the proton attached to carbon #6 of cholesterol esters, respectively. The resonance at 4.6 ppm is from cholesteryl esters and the resonance at 4.0 ppm is from wax esters (Figure 1). The resonances near 5.1 ppm have been assigned to squalene [29].

**Figure 2:**
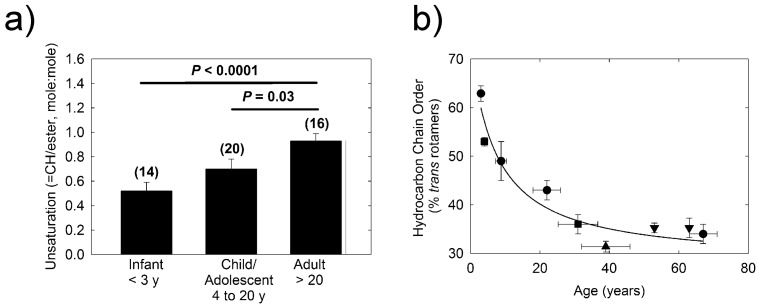
(**a**) Average hydrocarbon chain double bond content of meibum calculated from ^1^H-NMR spectra. Numbers in parenthesis are the number of samples; and (**b**) Lipid order (gel) to disorder (liquid crystal) phase transition parameters of meibum. Recalculated from (see text): (●) Reference [27]; (■) Reference [1]; (▲) this study; (▼) and Reference [26]. (▬) Curve fit to data using the parameter, hyperbola, hyperbolic decay equation: *f* = *y*_0_ + (*a* × *b*)/(*b* + *x*). All donors were normal and did not have signs or symptoms of dry eye. Data are average ± the standard error of the mean.

The total level of double bonds from cholesteryl and wax =CH resonances (5.32 and 5.35 ppm) relative to the sum of wax (4.0 ppm) and cholesteryl ester (4.6 ppm) resonances increased significantly (*p* = 0.03) from 1.0 ± 0.1 for infants to 1.4 ± 0.1 in children. Unsaturation of human meibum increased with age and the relative level of hydrocarbon *cis* =CH unsaturation of infants was significantly lower (*p* < 0.0001) compared with that of adults (Figure 2a). Samples above 20 years of age were grouped together based on the developmental Tanner stage and changes in blink rate, free fatty acids and meibum lipid phase transition parameters (see Discussion).

### 2.3. Infrared Spectroscopy

**Figure 3:**
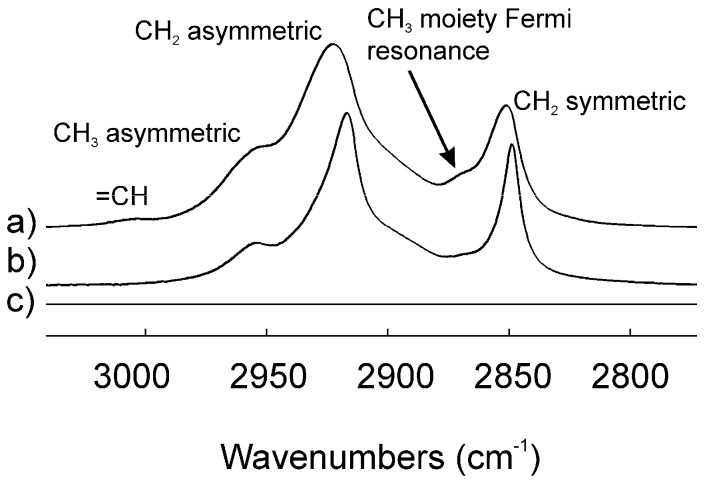
Infrared CH stretching region for: (**a**) a pool of meibum lipids from four normal adult donors; (**b**) meibum lipids from the same pool that was catalytically hydrogenated and (**c**) AgCl window.

**Figure 4:**
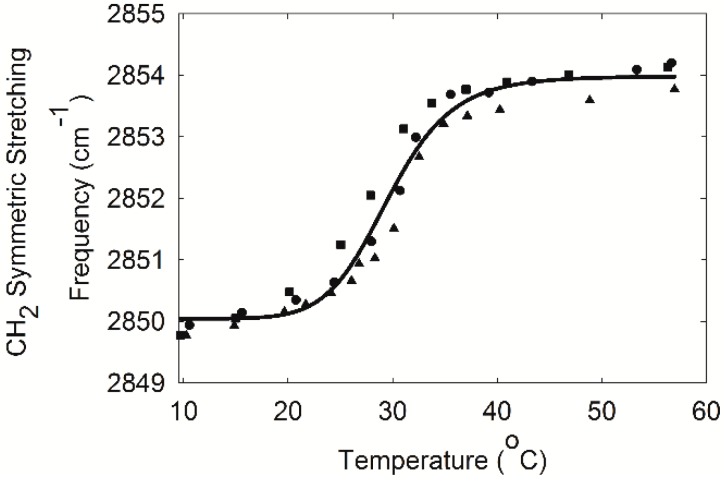
Lipid phase transition of meibum pooled from six Caucasian females 23, 24, 28, 32, 66, and 68 years old; a 29-year-old Caucasian male; and an 32-year-old African American female. The CH_2_ symmetric stretching frequency is related to lipid structural order. The higher the value for the frequency, the more disordered the lipid. Symbols are different trials. Symbols are for different trials.

Infrared spectroscopy was used to study lipid–lipid interactions and composition. The CH_2_ stretching and bending bands are predominant in the infrared spectra of lipids due to the large number of CH_2_ groups in their hydrocarbon chains. The CH stretching region of meibum is composed of five major bands (Figure 3) [26]. Note the catalytically hydrogenated sample has no =CH stretching band (Figure 3b). In this study, we used the frequency of the symmetric CH_2_ stretching band near 2850 cm^−1^ (*ṽ*_sym_) to estimate the *trans* to *gauche* rotamer content of the hydrocarbon chains. The *ṽ*_sym_ increased with an increase in temperature and the number of *gauche* rotamers, concurrent with a decrease in intensity (Figure 4) [26,30]. The peak height of the CH_2_ symmetric stretching band at 9 °C was approximately 0.23 absorbance units. The absolute intensity of the CH stretching region decreased by about 20% with an increase in temperature from 9 to 65 °C which was attributed partially to a 50% decrease in the CH_2_ symmetric stretching band [30]. A sigmoidal equation was used to fit and quantify the lipid phase transitions [26]. Two of the fitted parameters, the minimum and maximum *ṽ*_sym_, correspond to the most ordered and disordered states of hydrocarbon chains, respectively. Another fitted parameter was the phase transition temperature, which is the temperature at which half of the lipid molecules undergo a phase change. The fourth fitted parameter was the relative cooperativity of the phase transition that describes how the order of a lipid influences that of neighboring lipids. Broad phase transitions have a relatively smaller absolute value of the cooperativity. Lipid phase transition parameters for a pool of human meibum used in the saturation study are listed in Table 1. Lipid order was measured close to the surface temperature of the human eye, 33.4 °C, by extrapolating the *ṽ*_sym_ at 33.4 °C from the fit of the phase transition and then converting *ṽ*_sym_ to the percentage of *trans* rotamers [26]. The lipid order measured in this study, (31 ± 2) % *trans* rotamers), reinforced the correlation between a decrease in lipid order with increasing age (Figure 2b, *r* = 0.963, *p* < 0.01).

**Figure 5:**
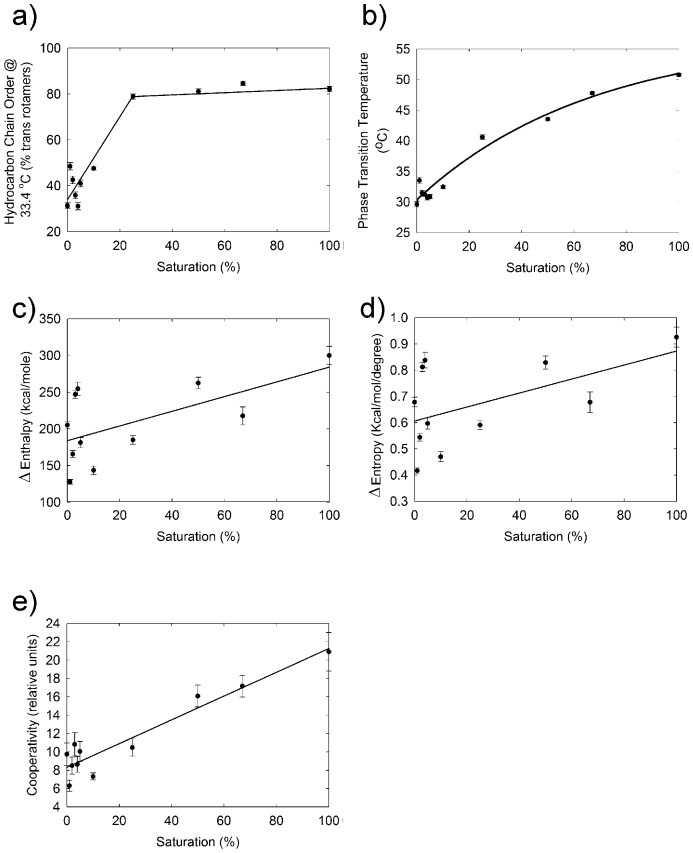
Lipid phase transition parameters for human meibum from Figure 4 that was mixed with catalytically hydrogenated meibum from the same pool. The percent saturation is the percentage of catalytically saturated meibum mixed with normal meibum. Data are average ± the standard error of the mean and reflect the experimental error for one sample. (▬) Best fit, linear regression (**a**,**c**–**e**) with a line order of 1 and (**b**) linear regression with a line order of 3.

Meibum lipid was catalytically saturated and the lipid phase transition parameters were measured. Lipid order at 33.4 °C increased significantly (*p* < 0.0001) from 39 ± 3% to 82 ± 1% between 0% and 25% saturation (Figure 5a). Above 25% saturation, lipid order reached a maximum. The lipid phase transition temperature for meibum lipids increased significantly (*p* < 0.01, *r* = 0.963) with saturation, as expected, from about 30 to 51 °C (Figure 5b). The change in enthalpy (∆H) (Figure 5c), and entropy (∆S) (Figure 5d), and cooperativity (Figure 5e) of the lipid phase transition increased substantially with saturation, *p* < 0.01, 0.05, and 0.1; and *r* = 0.626, 0.544, and 0.950, respectively.

**Figure 6:**
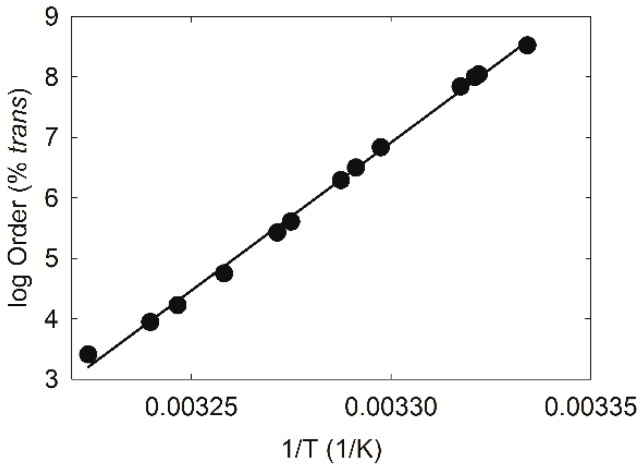
A typical Arrhenius plot for a pool of human meibum used for the saturation study. The slope of the line is used to calculate the changes in enthalpy (∆H) and entropy (∆S) of the lipid phase transition.

**Figure 7:**
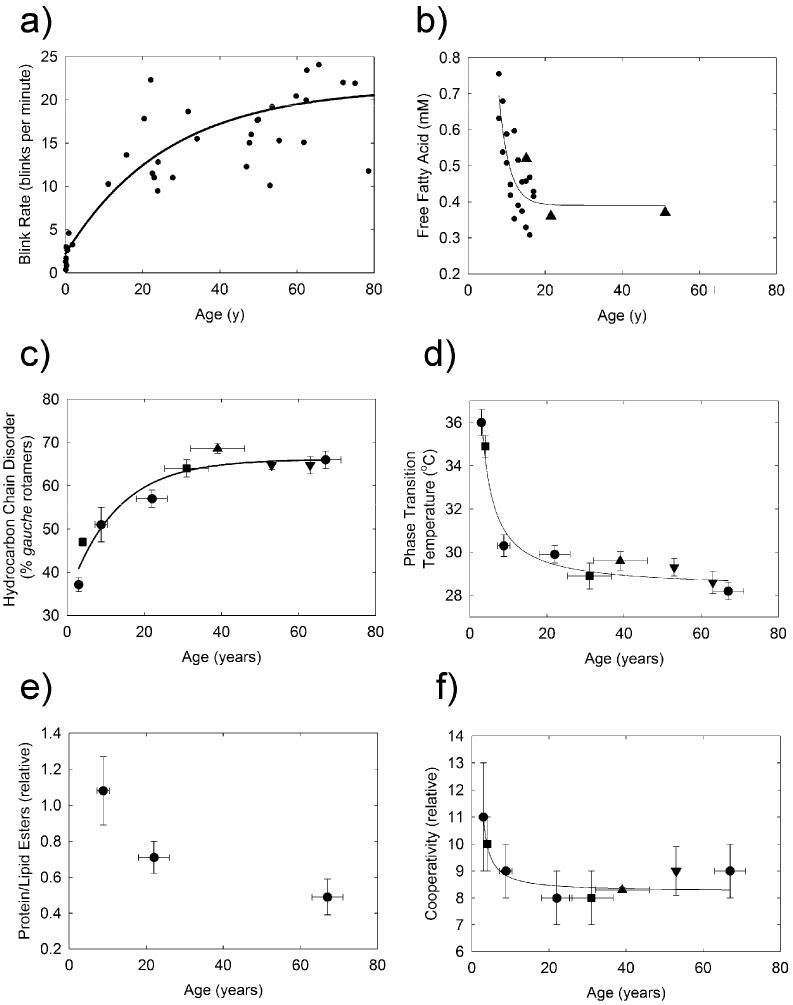
(**a**) From References [5,8,14,15,35,36,37,38,39,40,41,42,43,44,45,46,47,48,49,50,51]; (**b**) (▲) from Reference [52] and (●) Reference [53]; (**c**,**d**,**f**) hydrocarbon chain order at 33.4 ^°^C and phase transition parameters for the lipid order (gel) to disorder (liquid crystal) phase transition of human meibum. (●) Reference [26]; (■) Reference [1]; (▲) this study and (▼) Reference [25]. (▬) Curve fit to data using the parameter, hyperbola, hyperbolic decay equation: *f* = *y*_0_ + (*a* × *b*)/(*b* + *x*); (**e**) Data from Reference [54]. All donors were normal and did not have signs or symptoms of dry eye. Data are average ± the standard error of the mean.

Arrhenius plots used to calculate the ∆H and ∆S values from the lipid phase transitions were linear, with correlation coefficients greater than 0.998 (Figure 6). For comparison of phase transition parameters of catalytically saturated meibum with age related changes, we refitted the phase transition curves from previous studies and recalculated the percent *trans* rotamers because in previous publications [1,25,26,27], the Equation used to curve fit the phase transitions was a general equation for sigmoidal curves. Equation (1) used in the current study is more physiologically relevant as it is related to the “Hill” Equation used to measure enzyme kinetics. Another reason to recalculate the previously measured phase transitions is that the minimum and maximum *ṽ*_sym_ used in the older studies were less accurate. In studies before our 2007 study [26], the maximum *ṽ*_sym_ of 2854.5 cm^−1^ was estimated from phosphatidylcholine in CHCl_3_. In this study, we used a maximum *ṽ*_sym_ of 2855.36 cm^−1^ calculated from an isomeric distribution of hexanes [26]. In addition, in previous studies, the minimum *ṽ*_sym_ of 2849 cm^−1^ was estimated from dipalmitoylphosphatidylcholine at −20 °C. In this study, we used a minimum *ṽ*_sym_ of 2848.00 cm^−1^ calculated from distearoylphosphatidylcholine at −50 °C [26]. Data using the parameters in citation 26 are plotted in Figure 2a and Figure 7c,d,f.

## 3. Discussion

A major finding of this study is that human meibum lipid hydrocarbon chain unsaturation increases with age in agreement with previous FTIR [27], and NMR [23] spectroscopic studies. However, a greater number of samples, 69, were measured in the current study and, in previous studies, a 500 MHz NMR spectrometer was used and the resonance from the =CH of cholesterol was not resolved from the =CH due to the hydrocarbon *cis* =CH resonance. The contribution of the =CH resonance of cholesterol was significant, 20% of the total intensity of =CH resonances. In the current study, we used a 700 MHz NMR spectrometer that allowed the resolution of the two resonances thus circumventing this shortfall. In the current study, unsaturation was related to the amount of wax and cholesteryl esters. This is more meaningful and accurate than relating saturation to the intensity of all the resonances as in the previous study. Furthermore, in the previous study [24], the resonance at 1.39 ppm was the major resonance in the NMR spectra and was from protonated *h*-cyclohexane, a contaminant of the *d*-cyclohexane that was incorrectly assigned to the meibum lipid CH_2_ resonance. In this study, we used CDCl_3_ to circumvent this issue.

Increasing human meibum hydrocarbon chain unsaturation with age (Figure 2a) was related to hydrocarbon chain order (fluidity, Figure 7c), and a significant decrease in cooperativity (Figure 7f, *r* = 0.940, *p* < 0.01), and the phase transition temperature (Figure 7d, *r* = 0.982, *p* < 0.01). The change in these parameters was most dramatic between 1 and 20 years of age. The significant decrease in the phase transition parameters between 1 and 20 years of age can be explained by the observation that the phase transition temperature is linearly related to meibum lipid order [54]. The largest decline in the meibum phase transition temperature and hence the largest decline in lipid order occurred between 1 and 20 years of age (Figure 2b and Figure 7d). The change in blink rate with age (Figure 7a) was closely related with the increase in hydrocarbon chain disorder (Figure 7c), decrease in the plasma levels of free fatty acids (Figure 7b), phase transition temperature (Figure 7d) and cooperativity (Figure 7f). Correlation does not necessitate cause, but it is interesting that the breaks in the curves in Figure 7 occur around 20 years of age, at a Tanner stage V and adult level of development [55]. It is reasonable to speculate that endocrine changes with adolescence could be responsible for the observed break in the curves since the metabolism of lipids is under hormonal control.

Our catalytic saturation study showed that meibum hydrocarbon chain saturation was directly related to lipid order, phase transition temperature, cooperativity, ∆H and ∆S. Saturated hydrocarbon chains contain more *trans* rotamers and pack much more tightly than unsaturated hydrocarbon chains due to bends introduced into the hydrocarbon chains from the *cis* C=C bond. As the saturated hydrocarbon chains containing more *trans* rotamers pack more tightly together compared with hydrocarbon chains containing *cis* C=C bonds, it takes more enthalpy to break the van der Waal’s interactions between the saturated hydrocarbon chains, thus the ∆H of the lipid phase transition is greater for saturated hydrocarbon chains compared with unsaturated chains. As expected, a 40% increase in saturation from adult meibum to meibum from infants (Figure 2a) would be expected to increase the phase transition of adult meibum from 28 to 40 °C, similar to the observed increase from 28 to 36 °C, (Figure 7d). Our catalytic hydrogenation study also showed that the saturation driven increase in the phase transition temperature (Figure 5b) could account for an increase in lipid hydrocarbon chain order (Figure 5a) from about 30% in adults to about 80% for infants, a little more than the 60% order observed for infants (Figure 2b). Other factors such as hydrocarbon chain branching and hydroxyl groups could contribute to disordering meibum [34], whereas protein (Figure 7e) could contribute to the ordering of meibum [32,56]. Saturation correlated with the phase transition temperature of pure and native membranes and may contribute to lipid order more than phospholipid, wax, cholesteryl ester content or hydrocarbon chain length or branching, [54].

The lipid phase transition temperature and cooperativity measured by FTIR in this work were reasonably close to those measured in our previous FTIR study and those of others using different techniques (Table 1), especially considering that the age, race and gender of the samples were not exact. The value we obtained for the ∆H of the meibum lipid phase transition is much larger than that reported using differential scanning calorimetry (DSC, Table 1). The reason for this difference may be due to technical differences or because DSC measures the total ∆H of the phase transition which includes the ∆H of hydrocarbon and interface interactions whereas the ∆H reported in the current study measures the ∆H for the transition of a mole of *trans* rotamers to a mole of *gauche* rotamers. There may be about eight *trans* rotamers per hydrocarbon chain. From the maximum and minimum infrared *ṽ*_sym_ of the phase transition, we calculate that in the ordered “gel phase” at low temperature, 72% of the rotamers are *trans* and 18% of the rotamers are *trans* in the disordered “liquid crystal phase” at higher temperature. Therefore, we estimate that DSC measures the ∆H for only 53% of the total isomers, the ones that undergo a *trans* to *gauche* change. Because the hydrocarbon chains are not completely ordered (solid) below the phase transition temperature and not completely disordered (liquid) above the phase transition temperature, the transition is called a gel to liquid crystalline phase transition and not a melting. Meibum compositional differences in hydrocarbon chain saturation can account for meibum structural differences with age as suggested in the current study. Lipid saturation [56], order and phase transition temperature [54] are higher in donors with Meibomian gland dysfunction compared with adults. Intuitively, meibum should be ordered enough to flow out of the Meibomian glands and fluid enough to spread on the surface of the tears. The relationships between meibum lipid structure and tear film function are less clear with dry eye that they are with age. The hydrocarbon chain order and phase transition temperature of meibum from donors with dry eye and unstable tears is 49% *trans* and 28 °C, respectively, comparable to that of donors younger than 10 years old with extremely stable tears, 50% to 60% *trans* and 35 °C, respectively. Therefore, other factors in addition to meibum lipid structure such as elevated levels of protein [32,56], cooperative unit size [33], loss of squalene [29], inflammation [57], sebum [1], interactions between meibum and moieties in tears [58,59,60], differences between the lipid composition of tears and meibum [1,26,58,60], and aqueous deficiency could all contribute to functional derangements with dry eye. Future studies focused on the role of meibum structure and tear film function are needed.

The infrared spectroscopic parameters discussed above are relevant to bulk meibum in the Meibomian gland and on the surface of the eye lid. The change in structural order of meibum with age could also be related to the structural order of meibum on the surface of tears since most (94%) of the lipid on the tear film surface is not in contact with the aqueous interface. However, we used Langmuir trough technology to measure how saturation influenced the surface properties of meibum [25] and compared native meibum with meibum that was 100% saturated, a level that was not physiological. We have completed a study comparing the rheology of meibum at physiological saturation levels for comparison with the composition, structure and functional data from the current study. We may speculate that the functional consequence of a more ordered, more elastic saturated meibum as observed for infants and the higher maximum surface pressure observed in pressure area curves of saturated meibum compared to native meibum suggests that more saturated meibum films could be more stable, especially under the high shear stress of a blink [25].

## 4. Experimental Section

### 4.1. Materials

Silver chloride windows for infrared spectroscopy were obtained from Crystran Limited, Poole, UK. Platinum (IV) oxide was obtained from the Sigma Chemical Company (St. Louis, MO, USA).

### 4.2. Diagnosis of Normal Status

Normal status was assigned when the patient’s Meibomian gland orifices showed no evidence of keratinization or plugging with turbid or thickened secretions and no dilated blood vessels were observed on the eyelid margin. Normal donors did not recall having dry eye symptoms. Written informed consent was obtained from all donors and protocols and procedures were approved by the University of Louisville Institutional Review Board # 11.0319, August 2016. All procedures were in accord with the Declaration of Helsinki.

### 4.3. Collection and Extraction of Lipid from Meibum

Meibum lipid was expressed from the eye lids [61] and was collected with a platinum spatula with attention to avoiding scraping of the eyelid margin. Donors had no signs or symptoms of dry eye. Expressed meibum was dissolved in 1.5 mL CDCl_3_. The samples were pooled for catalytic hydrogenation.

### 4.4. Catalytic Hydrogenation

Half the pooled meibum was decanted to be catalytically hydrogenated. Saturated meibum was prepared as we did for sphingomyelin [26,62,63]. Platinum (IV) oxide (7.4 mg) was used as a catalyst to reduce the samples with hydrogen at room temperature and atmospheric pressure for approximately 4 h with stirring. Centrifugation was used to separate the catalyst from the solution. Catalytically saturated samples were quantitatively mixed with sample that was not catalytically saturated to provide mixtures containing 1%, 2%, 3%, 4%, 5%, 10%, 25%, 50%, and 67% of catalytically saturated meibum.

### 4.5. Saturation Analysis Using H-NMR

On the day of NMR measurement, the sample was sonicated under an atmosphere of argon gas in an ultrasonic bath (Branson 1510, Branson Ultrasonics, Danbury, CT, USA) for 10 min and placed into a NMR tube for spectral measurement. Meibum-CDCl_3_ samples were transferred from the microvial to a NMR tube using a glass pipet. Spectral data were acquired using a Varian VNMRS 700 MHz NMR spectrometer (Varian, Lexington, MA, USA) equipped with a 5-mm ^1^H{^13^C/^15^N}^13^C enhanced pulse-field gradient cold probe (Palo Alto, CA, USA). Spectra were acquired with a minimum of 250 scans, 45° pulse width, and a relaxation delay of 1.000 s. All spectra were obtained at 25 °C. Spectra were processed and integration of spectral bands was performed with GRAMS/386 software (Galactic Industries, Salem, NH, USA).

To quantify the relative level of *cis* hydrocarbon =CH (5.32 ppm) bonds, the intensity of the =CH resonance from cholesteryl esters (5.35 ppm) was subtracted from the total area of the 5.32 and 5.35 ppm resonances then divided by the sum of the resonances from cholesteryl and wax esters at 4.6 and 4.1 ppm, respectively.

### 4.6. Measurement of Lipid Phase Transitions Using FTIR Spectroscopy

Lipid phase transitions were measured as described previously [25]. About 500 µL of sample in CDCl_3_ was applied to a AgCl infrared window. The solvent was evaporated under a stream of Argon gas and the window was placed in a lyophilizer for 4 h to remove all traces of solvent. A Fourier transform infrared spectrometer (Nicolet 5000 Magna Series; Thermo Fisher Scientific, Inc., Waltham MA, USA) was used to measure the infrared spectra of the lipid on a AgCl window. The window was placed in a temperature-controlled infrared cell. The sample temperature was adjusted by an insulated water coil connected to a circulating water bath (model R-134A; Neslab Instruments, Newton, MA, USA) surrounding the cell. A thermistor touching the sample cell window was used to measure the sample temperature. The sample was cooled or heated at a rate of 1 °C/15 min. Temperatures were maintained within ±0.01 °C. Exactly 100 interferograms were recorded and averaged. Spectral resolution was set to 1.0 cm^−1^. Infrared data analysis was then performed (GRAMS/386 software; Galactic Industries, Salem, NH, USA). *ṽ*_sym_ was used to estimate the content of *trans* and *gauche* rotamers in the hydrocarbon chains. The OH–CH_2_ stretching region of the spectra was baselined between 3500 and 2700 cm^−1^. *ṽ*_sym_ was calculated from the center of mass of the CH symmetric stretching band by integrating the top 10% of the intensity of the band. The baseline for integrating the top 10% of the intensity of the band was parallel to the OH–CH region baseline. The change in *ṽ*_sym_ versus temperature was used to characterize lipid phase transitions as described previously [25]. Since rotamers are in either *trans* or *gauche* conformations, phase transitions were fit to a two-state sigmoidal equation using Sigma plot 10 software (Systat Software, Inc. Chicago, IL, USA):

*ṽ*_sym_ = (*ṽ*_sym_)_minimum_ + ((*ṽ*_sym_)_maximum_ − (*ṽ*_sym_)_minimum_)/(1 + (temperature/*T*c)^hillslope^) (1)

*ṽ*_sym_ is the frequency of the symmetric CH_2_ stretching band near 2850 cm^−1^. *T*c is the phase transition temperature.

*ṽ*_sym_ at 33.4 °C was extrapolated from the fit of the phase transition and then converting to lipid order which is the percentage of *trans* rotamers [25]. ∆H and ∆S were calculated from the slopes of Arrhenius plots [25].

### 4.7. Statistics

Curves were fit using Sigma plot 10 software (Systat Software, Inc., Chicago, IL, USA) and the confidence levels, *p*, were obtained from a critical value table of the Pearson product–moment correlation coefficient. A value of *p* < 0.05 was considered statistically significant. Error bars are the standard error of the mean.

## 5. Conclusions

Hydrocarbon chain saturation was directly related to lipid order, phase transition temperature, cooperativity, changes in enthalpy (∆H) and entropy (∆S) and could account for the changes in the lipid phase transition parameters observed with age. Unsaturation could contribute to decreased tear film stability with age.

## Figures and Tables

**Table 1:** Meibum Lipid Phase Transition Parameters.

Parameter	This Study FTIR	FTIR [1]	Microscopy [31]	Birefringence [32]	DSC [33]	Light Reflectance [34]
Hydrocarbon Chain Order (% *trans*)	31 ± 2	36 ± 2				
Phase Transition Temperature (°C)	29.6 ± 0.4	28.9 ± 0.6	32.1 ± 0.1	32 ± 2	30.2 ± 0.1	32 ± 1
Cooperativity	10 ± 1	8 ± 1	19.3 ± 0.1	7 ± 1, 34 ± 10	16, 40, 100	
Change in enthalpy (Kcal/mol)	205 ± 5	196 ± 2			6 ± 1	
Change in entropy (Kcal/mol/deg)	0.68 ± 0.02	0.650 ± 0.008				
Age (years)	39 ± 7	31 ± 6	29, 50	53, 38 ± 4	50 ± 2	47
Sample demographics	1 pool of 8	1 pool of 7	1 pool of 2	1 donor and 1 pool of 4	1 pool of 2	1 pool of 5
